# Academy of Stomatology

**Published:** 1900-06

**Authors:** 

**Affiliations:** Academy of Stomatology


### Reports of Society Meetings.

### ACADEMY OF STOMATOLOGY.

A REGULAR meeting of the Academy of Stomatology was held on the evening of January 23, 1900, at the rooms of the Academy, 1731 Chestnut Street, Philadelphia, Professor E. T. Darby, President *pro tem.*, in the chair.

A paper entitled "Alloys for Filling Purposes" was read by Dr. A. P. Fellows, of Philadelphia.

(For Dr. Fellows's paper, see page 379.)

### DISCUSSION.

*Dr. Rice.*—I have a clipping from the *Dental Brief*, by Dr. Bogue, in answer to the question, Has the mercury in amalgam fillings a toxic effect? He replies, "I have not made any experiments at all upon the toxic effects of mercury in amalgam fillings, perhaps because most of the patients for whom I have had the honor of operating have not, during my time, been subjected to a dull red heat, which is about the temperature required to produce either of the two poisonous salts of mercury."

*Dr. Neall.*—I came to learn something, and I have learned something. The way the essayist has handled the old information about alloys is gratifying. The new points he has made are also gratifying. I do not know much about the making of alloys, although I have had some little experience in placing the amalgam filling after the alloy has been made. I find that with two persons using the same alloy the results will differ: one will produce a good, solid, homogeneous filling, with good edge-strength, while the other will have a mass which in time will break down and become almost porous. Therefore, the working of the alloy is important, and no matter how good an alloy may be, if not worked properly it will produce bad results. My idea has always been that the material should be mixed in the mortar, afterwards transferred to the hand, and there further mixed to a plastic mass. When amalgam creaks under palm manipulation, it is good. As to toxic effects of the mercury, I may say that I

inserted a number of amalgam fillings for a gentleman some years ago, and later he came back and said, "I have terrible distress. My physician says that it is on account of the mercury in the fillings." In working over the young man I had to turn away frequently on account of the odor of his breath. After questioning him at length I found that he was suffering from nervous dyspepsia. I said, "If you will go to your physician and allow him to treat you for this trouble, no doubt you will get well." And I said, also, "If you will send your physician to me and let him tell me what effect free mercury in a person's mouth has, I will learn something." The physician did not come, but the young man has been a patient of mine ever since. He is perfectly well. There is another point that I would like to hear discussed, and that is, What becomes of the mercury after the filling hardens? In one of the dental journals some time ago, one of our learned brethren said that it had an effect upon the system; that it became free afterwards in the mass and did its bad work. I have been taught, and I have seen from experiments of others, that when a filling has been properly mixed, prepared, and placed in, one week after, two weeks after, three weeks after, and months after, it has the same weight. It does not seem to decrease in weight at all. My idea of the subject is, that the mercury in the amalgam goes deeper into the filling, becomes a solid part of it, and that in an amalgam filling you will still have the same weight.

*Dr. Jack.*—Mr. President, I have very little to say on this subject in addition to what has already been stated.

In respect to the mercurial action of amalgam fillings upon the constitutional conditions of patients, much has been written, and experiments have been made with relation to the possibility of that action taking place. Probably some of you remember that in 1874, under Dr. Bogue's auspices, in connection with Dr. Hitchcock, of Boston, experiments were made with twelve varieties of amalgam then in common use, principally directed to the question raised in this discussion as to whether the amalgams can have any toxic action upon the human system. Specimens of each of these amalgams were placed in a quarter-ounce of saliva with seven drops of acetic, nitric, citric, and hydrochloric acid. That is four experiments for each formula. The whole forty-eight were then submitted to a water-bath kept at about 100° F. for over three months. The loss in weight was stated to be only about three grains in eleven pennyweights. Only citric acid appeared by these experiments to affect diminution in weight. The principal part of the loss was from the copper amalgam.

After the submission to heat and acids was completed the fluids of the test-bottles were joined together and submitted to Professor Chandler, who declared in his certificate that the samples of saliva contained no mercury in solution.

This would appear to settle the question. Dr. Bogue's paper will be found in Vol. VII., page 118, *Dental Cosmos.*

But there are circumstances and conditions under which amalgam fillings may produce constitutional effects, and these are, in short, that some amalgams are of such composition that they undergo electrolysis. I have observed fillings inserted with fulness, and after some years have found that they have lost substance. I have known some formulas of amalgam that I have used experimentally which in a few years were much reduced in quantity, showing that the fillings had been undergoing a process of electrolysis. This pertained to such as contained an excessive proportion of gold. If that action should be of rapid progress, one could readily admit that there could be some constitutional effect produced by the mercury or the metals compounded with or in association with mercury.

I might state, in some further elucidation of this subject and to illustrate my immediate remarks, that the copper amalgams undoubtedly had some influence upon constitutional conditions, for that was one of the results that occurred in the use of copper amalgam,—a very large proportion underwent a slow or rapid disintegration. Therefore, there might readily occur some constitutional disturbance. I had a patient at one time with several copper amalgam fillings who complained of disturbance of the throat, that had not occurred to her previously to their insertion. I removed them, and since then the disturbance has passed away. However, it is impossible for any one to establish the connection, because such a condition of the fauces might occur from other poisonous influences or constitutional conditions.

*Dr. Huey.*—I have, as you all have, been experimenting to a considerable extent with amalgams, and in years past have made a number of attempts to formulate an amalgam that would be better than any I could purchase in the shops. I must admit that I was not successful. As Dr. Flagg once said, It does not make so much difference what is put into the crucible as what is taken out of it, and I found that, no matter how carefully I weighed my ingredients, I always had a loss, and I was not enough of a chemist to ascertain what that loss was; but be that as it may, I have always been able to purchase a better amalgam for use than I could make myself.

There are one or two points that I have observed in the use of amalgam that I would like to speak of. In the first place, all amalgams are difficult to make homogeneous, especially when mixed in the mortar. Particles of the amalgam adhere to the sides of the mortar and are difficult to remove. I hit upon an expedient a good many years ago of mixing the amalgam in my hand and then finishing it on heavy calendered paper with a glass pestle. I found that after rolling up the amalgam in a ball, I could mix it on the paper pad in half a minute more thoroughly than in a mortar or in any other way. There is one disadvantage, it makes the amalgam set more quickly, but pack beautifully. In the matter of packing, I inserted amalgam fillings for a good many years without having any appreciation of the difficulty of packing them, and it was not until I was making the experiments I spoke of and packed the amalgam into glass tubes, to ascertain whether it would shrink or not, that I appreciated the difficulty of packing amalgam. It takes very much more time than we commonly give to the insertion of amalgam fillings. After packing and filling in the ordinary manner in a glass tube, it is wonderful how many air-spaces exist between the filling and the tube, and how far a coloring fluid, introduced into the tube, percolates through these air-spaces. I then began to use the electric mallet in packing amalgam fillings, and do so now. After packing I rub off the surplus of mercury with bibulous paper, and pack again and again, until I make a fairly hard surface and build to the desired shape.

*Dr. Jack.*—If I may be allowed, I would like to say a word in support of what Dr. Huey has said. My method of mixing amalgams has been to do it in the mortar, for the reason that this method prevents admixture with the amalgam of water and oil of the skin as in the palm mix. After amalgamation in the mortar, I take the mass out, roll it and knead it in a napkin, to produce homogeneity.

In regard to inserting amalgams, my course is to pack them with the automatic mallet. This is similar to the method adopted by Dr. Huey. That method produces what I believe to be a sufficient solidity and adaptation.

*Dr. William Trueman.*—There is necessarily a difference in amalgamating and inserting amalgams due to the formula. I think we should insist on knowing what the alloy is, and not use it blindly, as we are compelled to do when this information is withheld. I think the time has come when, in justice to our patients and our own reputation, we should insist on knowing the formula, at least sufficiently to enable us to use judgment as to how and when to use it.

Copper is an exceedingly treacherous metal to use in alloy where color is important. I was very much impressed, when spending an afternoon with Dr. Flagg some time ago, at his reference to copper. In conversation I mentioned a formula I had been using, and he said, "That is all right, but the copper will discolor." I said, "Why, doctor, that is its strong point,—it does not discolor." He said, "How long have you been using it?" I said, "Four or five years." He said, "I will give you two more to change your mind." He proved a true prophet,—I have changed my mind. I was very much surprised to learn that copper was an exceedingly treacherous metal,—only, however, where color is concerned. It gives the amalgam a much finer grain; it controls contraction and expansion; it is a reliable tooth preserver, and can be so graded that the filling may be finished up very nicely when first polished, and retains its color a long time; but I found that in six or seven years the fillings invariably tarnished, and sometimes became very dark.

In regard to aging, I think that is a very important matter. I had a peculiar experience some time ago. I made up a batch of alloy by a new formula, and to anneal it placed it in the oven of the kitchen range and gave instructions that it should be taken out in the morning before getting breakfast. It so happened that the fire burned up in the night more than I expected. When I came down in the morning they had a good fire, and I found my alloy still in the oven and it was pretty hot. Just after cutting it was a very quick setter,—even in a few minutes it became unworkable. After the heating it took about twenty-four hours to set. After some weeks it set hard in a few hours' time. I am accustomed to anneal my alloy when the furnace is going in the winter time, and put it in the heating chamber when there is a moderate fire, letting it remain about a couple of days. It is not scientific, but very satisfactory.

The doctor, in speaking of making the alloy, referred to the difficulty of getting it thoroughly homogeneous. For some time I have been using, virtually, Dr. Flagg's plan. First fuse in the crucible enough borax to thoroughly cover the alloy. Not only should the crucible be lined with borax, but there should be enough at the bottom so that when the alloy is all in it is thoroughly covered; that may mean about an inch or an inch and a half deep when the borax is thoroughly vitrified. Increase the heat until this is as clear and limpid as so much water, then add the tin. It at once melts and sinks under the borax, where it is secure from oxidation. Now add the gold, platinum, and copper, rolled very thin; then the silver; stir it well with a clay pipe-stem, add any volatile metal, such as zinc, and immediately pour into the ingot. Dr. Flagg, I believe, prefers to commingle the metals and pour them together into the crucible as soon as the borax has attained the stage of thorough vitrifaction. The important point is to first get the borax well fused, and to use plenty of it. Oxidation is thus prevented. The melt leaves the crucible perfectly clean. The loss in making five or ten ounces by this method is very trifling; if there is no loss from "sputtering," it may not amount to a grain. Always break the crucible after pouring; the temptation is strong to use it again when we see it so nicely glazed, and so clean; the borax has a strong affinity for the material of the crucible, and invariably, when used a second time, the alloy goes through the bottom.

I prefer to comminute the alloy in a turning-lathe, and aim to get a thin, mat surface shaving. A planer or a shaping-machine does this better than the lathe. With a tin-silver alloy, the shaving will be well defined; if silver is the dominant metal, the alloy being hard and brittle, it breaks up into minute flakes. In either form I find that it takes the mercury much more readily than when reduced by the file. Although the tool is advanced very slowly, I find the turning-lathe more rapid and less fatiguing than the file.

In our gold work we find it desirable to use cohesive and non-cohesive gold. I think, to satisfactorily meet all cases requiring its use, that we need more than one formula of alloy. My preference is for amalgam containing a large percentage of tin. I have been making it for over forty years, and have been using it for nearly that length of time to my own satisfaction. To get the best result it must be used dry,—mixed and ground up with the mercury and the surplus pressed out, so that when it is packed in the cavity, although it is not, as we say, set, it is pretty nearly as hard as it can be made. It is difficult to use it in this condition for contour work, where we have not good walls, unless a matrix is available.

When I want to contour, I prefer to have an alloy containing a large percentage of silver. I have been very strongly impressed with the fact that where the percentage of silver is too high you cannot get thorough amalgamation.

The different treatments required to get the best from alloys containing a high percentage of silver, or a high percentage of tin, is not thoroughly appreciated. If you mix them both in the same way, and pack them in the same condition, you will have poor fillings in each case. Properly treated and used, either will make good reliable work.

*Dr. Hickman.*—The essayist reminds me of an experience that I had with amalgam about two years ago. Some one introduced an imitation of the original Felllowship alloy, and they thought at first it was just as good as the Fellowship. A number of us took it up. It was somewhat cheaper, something new, so we started in to use it, and instead of getting hard, it proved a difficult-setting amalgam; it would get hard enough to polish, but in the course of a few days one could take an excavator and cut it out. I do not know whether that was due to the amount of zinc in the alloy, or what it was, but it taught a good many of us a lesson about going in diverse ways and using amalgam we did not know anything about.

*Dr. Huey.*—Allusion has been made to the Fellowship alloy. I have been using it and the Twentieth Century alloy, and have found them better than anything I have ever tried. They work so much alike that it is difficult for me to see any difference. It would be interesting to know if there are any new alloys, and it would be pleasant to hear the experience of those using alloys as to which are most successful.

*Dr. Register.*—I have never had any experience in making alloys for filling purposes. A number of years ago I was very much interested in finding out what metals changed their forms during crystallization, and to that end made an amalgam of mercury and gold and silver and tin and copper, each separately. I did not make one of zinc. I found that silver was the only one with mercury that crystallized, and the expansion was so great as to surprise me very much. At that time I was testing the alloys I used in a little plate of steel containing a number of holes with tapering sides. If after filling one of the holes, as I would fill a cavity, I could push it out from the opposite side, I judged that the amalgam would shrink; and I could find out if it expanded by running a rule on the surface. The experimental mixes of pure gold and pure tin amalgam were very plastic and not much harder at the end of a week than when first made. Pure copper amalgam was persistently granular. Pure silver amalgam mixed nicely with some crepitation, but it crystallized rather quickly and became very hard. It expanded perhaps twenty per cent. of its bulk. I tested all the alloys I used at that time in the little matrix, and also kept an accurate record of the mouths in which I used them. In looking up these records of each alloy, which go back for about eighteen years, I feel much disappointed in a number that stood the matrix test, but were short lived in the mouth, the alloy having the best record for a long time being the "Globe" alloy, though later on its record shows that it had not proved so serviceable, so I gave it up for that reason and have not used it for a number of years. A number of fillings, however, made of Globe alloy twelve to eighteeen years ago are doing good service to-day, but many have failed.

For the last three years or more I have been using Fellowship exclusively, to get results in the mouth in every service required. In compound cavities I use a very simple matrix made of very thin copper or German silver, anywhere from thirty-four to forty gauge. This ligated to the tooth by six to a dozen turns of a silk ligature permits close adaptation of the matrix to the cervical margin; it is also readily burnished to shape to form a knuckle contour, and permits mallet force or rotary burnishing with an engine burnisher, a means I prefer, as the heat generated temporarily softens the amalgam and consequently causes increased plasticity. My tests of Fellowship alloy are too recent for judgment of other than its fine working qualities. I commenced getting it in ten-ounce lots, and found that the last of the lot mixed better, there being less crepitation. Washing in alcohol removes oxidation and aids homogeneity. While we want our gold made fresh, I really think that there is an improvement in working qualities from aging. The thin copper may be obtained at Shannon's hardware-store, Philadelphia, or it may be made at any hardware-store.

*Dr. Fellows.*—As far as my experience has gone, I believe one per cent. of gold will give almost as good edge-strength, and will control color nearly as well, as five per cent. will do. I have also noticed that copper will replace gold in nearly every respect except color. I cannot agree that metals should be poured from one crucible to another, for when metals are heated to that extent, they are thoroughly incorporated, and if exposed to air while pouring, the tin and zinc at once will oxidize, and a part be lost. To keep the original quantity, do not expose it to the air.

An amalgam with too great an amount of tin, and possibly one containing too great an amount of zinc, would suffer more or less loss by attrition, a fact which may explain Dr. Jack's contention.

While pure copper amalgam is of questionable utility, alloys containing copper and gold together, as subordinate or modifying constituents, are very valuable. The latter combination prevents tarnishing.

Dr. Huey mentioned tube tests. They enable us to see whether our manner of packing is faulty. I do not believe one can pack amalgam well without force, either mallet or rotary. If our amalgam does not set too rapidly, and goes into the cavity in small pieces and is thoroughly packed, we will get a thorough filling and one which will fill the cavity absolutely, especially when a matrix reduces the compound cavity into a simple one. Dr. Trueman mentions copper modified amalgams, and claims that they will show discoloration in the course of from five to seven years. I have some fillings I have had in use for nearly fourteen years, and I cannot say they are affected in the least. Dr. Register mentioned the keeping of records. I think this is an excellent thing. I have a register of nearly every filling which I have put in, of whatever material, and when a patient returns to me after a number of years, I am able to see what kind of a filling serves best. I think that is the way we ought to study materials. He also mentioned the "Globe" alloy. Different makers of that alloy no doubt have produced different results, even in the same firm of manufacturers, just as different bakers make different bread from the same formula. All alloys brought into our offices to be put into teeth should be tested by us, and if not satisfactory, they should be returned. I have kept amalgams at times for some years, and have found a great deal better success in working them than when they were first made. I think it is probably due to the fact that the air acts upon the particles and oxidizes them.

*Dr. Cryer.*—Several gentlemen have spoken about packing the amalgam in glass tubes. I would recommend the roughening of the inside of the tubes, as it approximates more closely the conditions present in cleansed cavities in teeth. One can pack a filling much better, with closer adaptation to the walls, than with the smooth glass, and you will not have the penetration of analine coloring fluid between filling and tube wall nearly so great.

The same amalgam packed into smooth and roughened tubes can be pushed from the former when hard, while from the latter it cannot. The latter is, therefore, not a good test for shrinkage.

OTTO E. INGLIS,
*Editor Academy of Stomatology.*

